# Glutamine Supplementation Attenuates Expressions of Adhesion Molecules and Chemokine Receptors on T Cells in a Murine Model of Acute Colitis

**DOI:** 10.1155/2014/837107

**Published:** 2014-05-07

**Authors:** Yu-Chen Hou, Jin-Ming Wu, Ming-Yang Wang, Ming-Hsun Wu, Kuen-Yuan Chen, Sung-Ling Yeh, Ming-Tsan Lin

**Affiliations:** ^1^Department of Surgery, National Taiwan University Hospital, Taipei 100, Taiwan; ^2^School of Nutrition and Health Sciences, Taipei Medical University, Taipei 110, Taiwan; ^3^Department of Primary Care Medicine, College of Medicine, National Taiwan University, Taipei 100, Taiwan

## Abstract

*Background*. Migration of T cells into the colon plays a major role in the pathogenesis in inflammatory bowel disease. This study investigated the effects of glutamine (Gln) supplementation on chemokine receptors and adhesion molecules expressed by T cells in mice with dextran sulfate sodium- (DSS-) induced colitis. *Methods*. C57BL/6 mice were fed either a standard diet or a Gln diet replacing 25% of the total nitrogen. After being fed the diets for 5 days, half of the mice from both groups were given 1.5% DSS in drinking water to induce colitis. Mice were killed after 5 days of DSS exposure. *Results*. DSS colitis resulted in higher expression levels of P-selectin glycoprotein ligand- (PSGL-) 1, leukocyte function-associated antigen- (LFA-) 1, and C-C chemokine receptor type 9 (CCR9) by T helper (Th) and cytotoxic T (Tc) cells, and mRNA levels of endothelial adhesion molecules in colons were upregulated. Gln supplementation decreased expressions of PSGL-1, LFA-1, and CCR9 by Th cells. Colonic gene expressions of endothelial adhesion molecules were also lower in Gln-colitis mice. Histological finding showed that colon infiltrating Th cells were less in the DSS group with Gln administration. *Conclusions*. Gln supplementation may ameliorate the inflammation of colitis possibly via suppression of T cell migration.

## 1. Introduction

Inflammatory bowel disease (IBD), which includes Crohn's disease (CD) and ulcerative colitis (UC), is a relapsing and remitting disorder characterized by chronic inflammation of the gastrointestinal (GI) tract. The etiology of IBD remains unclear; however, both CD and UC are associated with enhanced leukocyte trafficking to the inflamed intestine [1, 2]. Previous studies indicated that effector T cells including CD4-positive T helper (Th) cells and CD8-positivecytotoxic T (Tc) cells play pivotal roles in the pathogenesis of mucosal lesions and chronic intestinal inflammation [3–5]. Blockage of lymphocyte migration to mucosal sites has therefore become a potential therapeutic strategy for IBD [6].

Lymphocytes migrate to specific tissues via a multistep process which is strictly regulated by adhesion molecules and chemokine receptors. Adhesion molecules present on the vascular endothelium and lymphocytes participate in the tethering, rolling, and adhesion of lymphocytes [7]. P-selectin and E-selectin appearing on activated endothelial cells interact with P-selectin glycoprotein ligand- (PSGL-) 1 expressed by lymphocytes, which promotes the initial tethering and subsequent rolling of lymphocytes over vessel walls. Integrins participate in rolling and firm adhesion of lymphocytes. Lymphocytes expressing *α*4*β*7 integrins roll on mucosal addressin cell adhesion molecule- (MAdCAM-) 1, which is required for homing of lymphocytes to intestinal sites [8]. Lymphocyte arrest mediated by integrins, such as lymphocyte function-associated antigen- (LFA-) 1 (also known as CD11a/CD18 and *αLβ*2 integrins) which interacts with their endothelial-cell ligands intercellular adhesion molecule- (ICAM-) 1, precedes extravasation into the underlying tissue [9].

Chemokines are small peptides which bind to chemokine receptors expressed on leukocytes and function as chemoattractants. They regulate lymphocyte homing to secondary lymphoid organs and transmigration into tissues by forming a chemokine concentration gradient which attracts lymphocytes to move towards an increasing concentration [10]. Dysregulation of chemokines and chemokine receptors was implicated in various autoimmune diseases, including IBD [11, 12]. Recent studies indicated that C-C chemokine receptor type 9 (CCR9) and *α*4*β*7 integrins are required for the localization of lymphocytes to the GI mucosa [13]. Agents developed for disrupting actions of CCR9 and *α*4*β*7 integrins showed promising results in IBD clinical trials [6].

Glutamine (Gln) is an immunomodulatory nutrient which is widely used in clinical practice [14]. Previous studies showed that Gln treatment has beneficial effects in different experimental models of colitis. Gln attenuates the expression of proinflammatory mediators [15] and improves outcomes which may be due to upregulation of heat shock proteins (HSPs) [16]. Recent work in our laboratory demonstrated that pretreatment with Gln suppresses cytokine expression of Th cells and ameliorates the severity of acute dextran sulfate sodium- (DSS-) induced colitis [17]. However, whether the beneficial effects of Gln are mediated by modulating lymphocyte trafficking in colitis is still unclear. Therefore, we investigated the influence of dietary Gln supplementation on T cell adhesion molecules and CCR9 expression in mice with DSS-induced acute colitis.

## 2. Materials and Methods

### 2.1. Study Protocols

Six-week-old male C57BL/6 mice were used in this study. Conventional mice were maintained in a temperature- and humidity-controlled room and were fed a standard chow diet ad libitum before the study. Care of laboratory animals was in full compliance with the* Guide for the Care and Use of Laboratory Animals* (National Research Council, 1996), and protocols were approved by the Institutional Animal Care and Use Committee of Taipei Medical University.

### 2.2. Experimental Design

**Figure 1:**
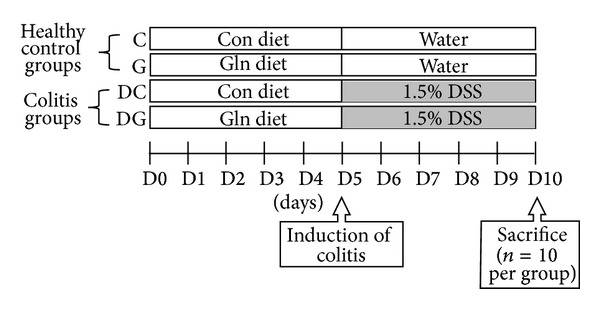
Flow diagram of the study design.

After 1 week of acclimation, 40 mice were randomly assigned to a control group or a Gln group in this study, with 20 mice in each group. Mice in the control group were fed a common semipurified diet, while the Gln group received a diet in which part of the casein was replaced with Gln. Gln provided 25% of the total amino acid nitrogen. This amount of Gln was proven to have an immunomodulatory effect in rodents [18–20]. Two diets were formulated to be isonitrogenous and isoenergetic (Table 1). After 5 d of being fed the diets, mice in the control and Gln groups were further divided into 2 respective subgroups. One subgroup was given distilled water, while the other subgroup received 1.5% (wt/vol) DSS (MW 40 kDa; MP Biomedicals, Solon, OH, USA) in the drinking water for 5 d to induce colitis. A flow diagram of the study design is shown in Figure 1. There were 4 groups in this study: control diet with distilled water (C group), Gln diet with distilled water (G group), control diet with DSS water (DC group), and Gln diet with DSS water (DG group). The respective experimental diets were given during the DSS exposure period. Body weights (BWs) were recorded daily, and all mice had free access to food and water throughout the study. At the end of the experiment, mice were anesthetized and sacrificed by cardiac puncture. Fresh blood samples were collected in heparinized tubes for measurements of the leukocyte population. Mesenteric lymph nodes (MLNs) were removed and processed for further analysis by flow cytometry. The colon was cut close to the ileocecal valve, and its length and weight were measured. Sections (1 cm) of the distal colon were cut. Colon tissues were fixed with buffered 4% paraformaldehyde for an immunohistochemical analysis.

### 2.3. Blood Leukocyte Distribution

**Figure 2:**
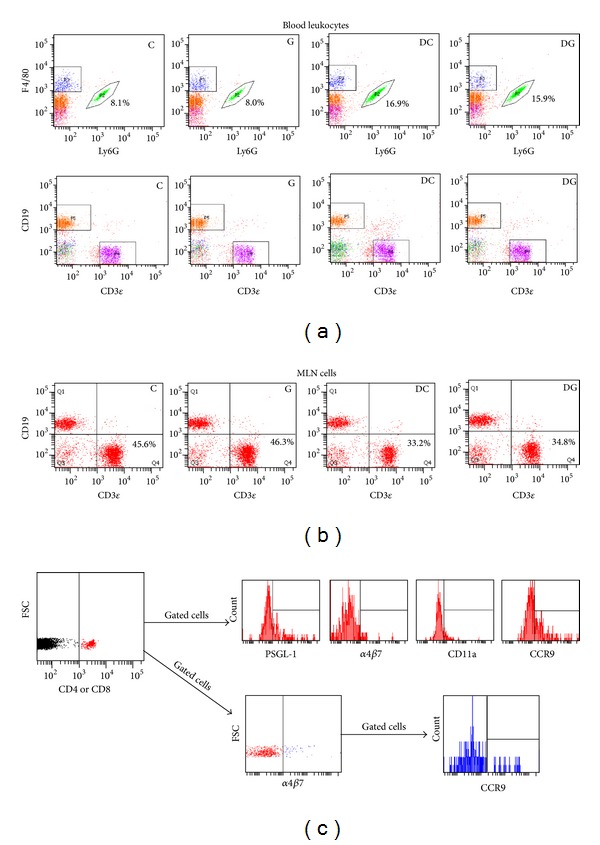
Representative flow cytometry plots. Blood leukocytes (a) were defined by gating on CD45-positive cells. The percentage of Ly6G-positive neutrophils from an individual representative mouse per group is listed. For analyzing the lymphocyte population in MLNs (b), MLN cells were first gated to exclude debris. Numbers indicate the percentage of CD3*ε*-positive lymphocytes in MLN cells. For analyzing the expression of adhesion molecules and chemokine receptors by T cells (c), lymphocytes were first identified based on low FSC and SSC characteristics. CD4- or CD8-positive lymphocytes were gated to analyze the percentages of adhesion molecule- and chemokine receptor-expressing CD4^+^ and CD8^+^ lymphocytes. Representative dot plots of leukocytes in blood are shown.

A five-color flow cytometric analysis was performed to determine the distribution of peripheral blood leukocytes. Antibodies against mouse leukocyte surface antigens were added to 100 *μ*L aliquots of whole blood. The antibodies used to detect different subsets of leukocytes were as follows: PerCP-conjugated anti-CD45 (Biolegend, San Diego, CA, USA) for leukocytes, PE-conjugated anti-F4/80 (eBioscience, San Diego, CA, USA) for monocytes/macrophages, FITC-conjugated anti-Ly6G (BD Biosciences, San Jose, CA, USA) for neutrophils, APC-conjugated anti-CD3*ε* (eBioscience) for T cells, and Pacific blue-conjugated anti-CD19 (Biolegend) for B cells. Antibodies were used at the concentration recommended by manufacturer. After a 30 min incubation at 4°C in the dark, red blood cells were lysed, and cells were suspended in staining buffer and then analyzed with a FACS Canto II flow cytometer (BD Biosciences). CD45-positive cells were gated, and results are presented as a percentage of specific CD-marker-expressing cells in blood leukocytes. Representative flow cytometry plots are shown in Figure 2(a).

### 2.4. Lymphocyte Populations in MLNs

Cell suspensions from MLNs were obtained by passing the tissues through a nylon cell strainer with a 40 *μ*m pore size (BD Biosciences) in RPMI1640 medium (Biological Industries, Kibbutz Beit Haemek, Israel). After centrifugation at 300 ×*g* for 10 min, pelleted MLN cells were suspended in 1 mL of staining buffer. One hundred microliters of cell suspension was incubated with APC-conjugated anti-CD3*ε* (eBioscience) and Pacific blue-conjugated anti-CD19 (Biolegend) for 30 min at 4°C in the dark. Stained cells were washed and resuspended in staining buffer to measure the lymphocyte population by flow cytometry. Percentages of T and B lymphocytes were determined by CD3*ε*- and CD19-expressing cells in MLN cells. Representative flow cytometry plots are shown in Figure 2(b).

### 2.5. Expressions of Adhesion Molecules and Chemokine Receptors by T Cells

Whole blood and MLNs were used to analyze adhesion molecule- and chemokine receptor-expressing T cells. Whole blood and MLN cells obtained as described above were split into 2 vials with 100 *μ*L in each aliquot, and these were incubated with Pacific blue-conjugated anti-CD4 (BD Biosciences) or Pacific blue-conjugated anti-CD8 antibodies (Biolegend). To investigate expressions of adhesion molecules and chemokine receptors, PE-conjugated anti-PSGL-1 antibodies (BD Biosciences), APC-conjugated anti-*α*4*β*7 integrin, FITC-conjugated anti-CD11a, and PerCP-Cy5.5-conjugated anti-CCR9 (Biolegend) were added. Stained cells were analyzed by five-color flow cytometry. Lymphocytes were gated on the basis of their forward- and side-scatter profiles. Fluorescence data were recorded, and results are presented as percentages of adhesion molecule- and chemokine receptor-expressing CD4^+^ and CD8^+^ lymphocytes. Representative flow cytometry plots are shown in Figure 2(c).

### 2.6. RNA Extraction and Real-Time PCR

Total RNA was isolated from colon tissue using the Trizol reagent (Invitrogen, Carlsbad, CA). RNA (1 *μ*g) was reverse-transcribed with a complementary (c)DNA synthesis kit (Fermentas, Glen Burnie, MD, USA) according to standard protocols. For real-time PCR, 5 *μ*L of 1/10 diluted cDNA was amplified in a 25 *μ*L PCR volume containing 12.5 *μ*L of 2X SYBR green master mix reagent (Applied Biosystems, Foster City, CA, USA). The reaction was performed with ABI 7300 Real-Time PCR System (Applied Biosystems) according to the thermocycling protocol recommended by the PCR system. Primer sequences were as follows: mouse ICAM-1 (5′-AGCACCTCCCCACCTACTTT-3′ and 5′-AGCTTGCACGACCCTTCTAA-3′), mouse P-selectin (5′-TCCAGGAAGCTCTGACGTACTTG-3′ and 5′-GCAGCGTTAGTGAAGACTCCGTAT-3′), mouse E-selectin (5′-TGAACTGAAGGGATCAAGAAGACT-3′ and 5′-GCCGAGGGACATCATCACAT-3′), and mouse 18S rRNA (5′-CGCGGTTCTATTTTGTTGGT3′ and 5′-AGTCGGCATCGTTTATGGTC-3′). All samples were analyzed in triplicate, and fold change for each target gene was calculated by the equation 2^−ΔΔ^Ct (ΔCt indicates the difference in threshold cycles between the test gene and 18S rRNA, and ΔΔCt indicates the difference of ΔCt between the experimental and C groups).

### 2.7. Immunofluorescence Staining

Double-staining combinations CD3-CD4 and CD3-CD8 were performed on 5 *μ*m paraffin-embedded colon sections. After antigen retrieval, sections were incubated with an antibody against CD3*ε* (Santa Cruz Biotechnology, Santa Cruz, CA, USA) overnight at 4°C and amplified with a rabbit anti-goat immunoglobulin G (IgG) secondary antibody conjugated with FITC (Santa Cruz Biotechnology). For colocalization, sections were then costained overnight at 4°C with secondary antibodies against CD4 (Abcam, Cambridge, UK) or CD8 (Novus Biologicals, Littleton, CO, USA) and amplified with the respective appropriate secondary antibodies: goat anti-mouse IgG or goat anti-rabbit IgG conjugated with rhodamine (Santa Cruz Biotechnology). Cell nuclei were counterstained with 4′,6-diamidino-2-phenylindole (DAPI, Sigma, St. Louis, MO, USA) for 10 min at room temperature. Digital images at 400x magnification per section were acquired using appropriate filters of a Zeiss Axiophot fluorescence microscope (Carl Zeiss MicroImaging LLC, Thornwood, NY, USA) fitted with a Nikon D1X digital camera (Tokyo, Japan). Cells containing both FITC and rhodamine labels appeared yellow. These images were then overlaid with DAPI-staining images to determine the infiltration of T lymphocyte subpopulations in the colon mucosa.

### 2.8. Statistical Analysis

All data are expressed as the mean ± standard error of the mean (SEM). Differences among groups were analyzed by an analysis of variance (ANOVA) with Tukey's test. A two-way ANOVA with Bonferroni correction was used to analyze differences in BW changes. A *P* value of <0.05 was considered statistically significant.

## 3. Results

### 3.1. BW and Weight/Length Ratio of the Colon

**Figure 3:**
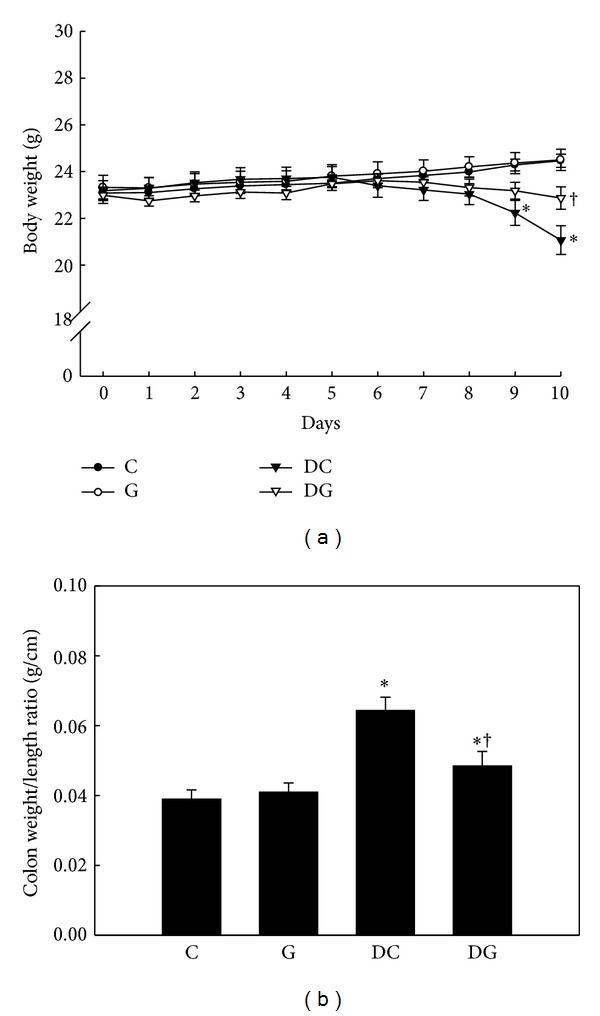
Body weight (a) and weight/length ratio of the colon (b). Data are presented as the mean ± SEM. C, normal mice fed the control diet; G, normal mice fed a glutamine-enriched diet; DC, DSS group fed the control diet; DG, DSS group fed a glutamine-enriched diet. *Significantly different from the C group (*P* < 0.05). ^†^Significantly different from the DC group (*P* < 0.05).

Initial BWs ranged 21~25 g and did not differ among the 4 groups. There was no significant difference in BWs during the study between the C and G groups. At 4 d (d 9) and 5 d (d 10) after DSS administration, weight loss was observed in the DC group compared to the C group, whereas mice with Gln supplementation maintained their BWs during the DSS exposure period. At the end of the study, BWs were significantly higher in the DG group than the DC group (Figure 3(a)). The weight/length ratio of the colon, an indicator of colonic edema, was significantly higher in the colitis groups than the C group (Figure 3(b)). Treatment with Gln attenuated colonic edema associated with DSS-induced inflammation.

### 3.2. Leukocyte Populations in Blood and MLNs

There was no significant difference in blood or MLN leukocyte subpopulations between the C and G groups. Compared to the C group, the DSS colitis groups had a higher percentage of blood neutrophils and lower T-cell population in MLNs. DSS exposure did not alter the blood monocyte and lymphocyte distributions. Also, subsets of effector T cells in the blood and MLNs did not change. Gln supplementation had no influence on blood leukocyte or MLN lymphocyte populations in normal or colitic mice (Table 2).

### 3.3. Adhesion Molecule and CCR9 Expressions by Th Cells

**Figure 4:**
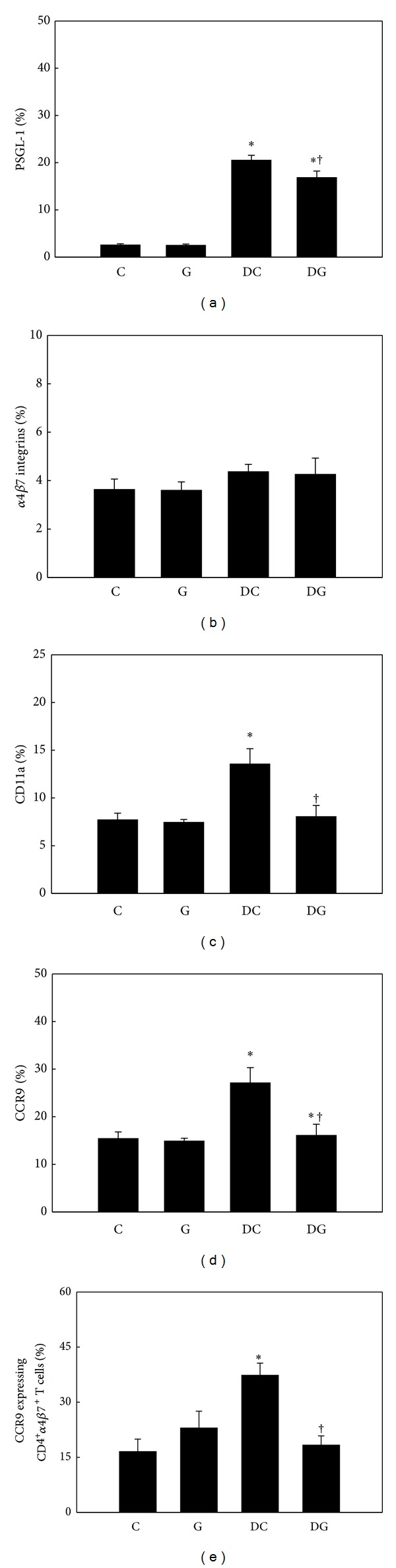
Percentage of adhesion molecule- and chemokine receptor-expressing T helper (Th) cells in blood. CD4-positive blood lymphocytes were gated to analyze expressions of PSGL-1, *α*4*β*7 integrins, CD11a, and CCR9 by flow cytometry ((a)–(d)). (e) Expression of CCR9 by CD4 and *α*4*β*7 integrin double-positive blood lymphocytes. Values are shown as the mean ± SEM. *Significantly different from the C group (*P* < 0.05). ^†^Significantly different from the DC group (*P* < 0.05).

**Figure 5:**
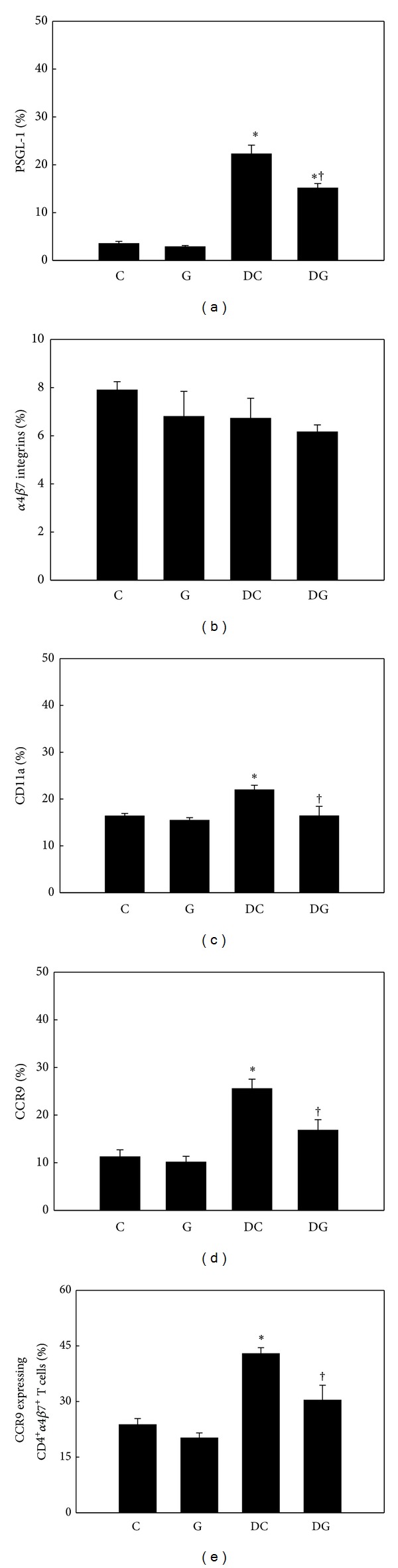
Percentage of adhesion molecule- and chemokine receptor-expressing T helper (Th) cells in mesenteric lymph nodes (MLNs). CD4-positive MLN lymphocytes were gated to analyze expressions of PSGL-1, *α*4*β*7 integrins, CD11a, and CCR9 by flow cytometry ((a)–(d)). (e) Expression of CCR9 by CD4 and *α*4*β*7 integrin double-positive MLN lymphocytes. Values are shown as the mean ± SEM. *Significantly different from the C group (*P* < 0.05). ^†^Significantly different from the DC group (*P* < 0.05).

Percentages of adhesion molecules and CCR9 expressed by blood and MLN Th cells did not differ between the C and G groups. DSS administration resulted in higher PSGL-1, CD11a (LFA-1 *α*L subunit), and CCR9 expressions by Th cells in both blood and MLNs, whereas no difference in *α*4*β*7 integrin expression was detected among the control and DSS groups. Mice in the DG group had lower percentages of PSGL-1-, CD11a-, and CCR9-expressing Th cells in blood (Figures 4(a)–4(d)) and MLNs (Figures 5(a)–5(d)). The expression level of CCR9 on *α*4*β*7-positive Th cells was also suppressed in the DG group (Figures 4(e) and 5(e)).

### 3.4. Adhesion Molecule and CCR9 Expressions by Tc Cells

**Figure 6:**
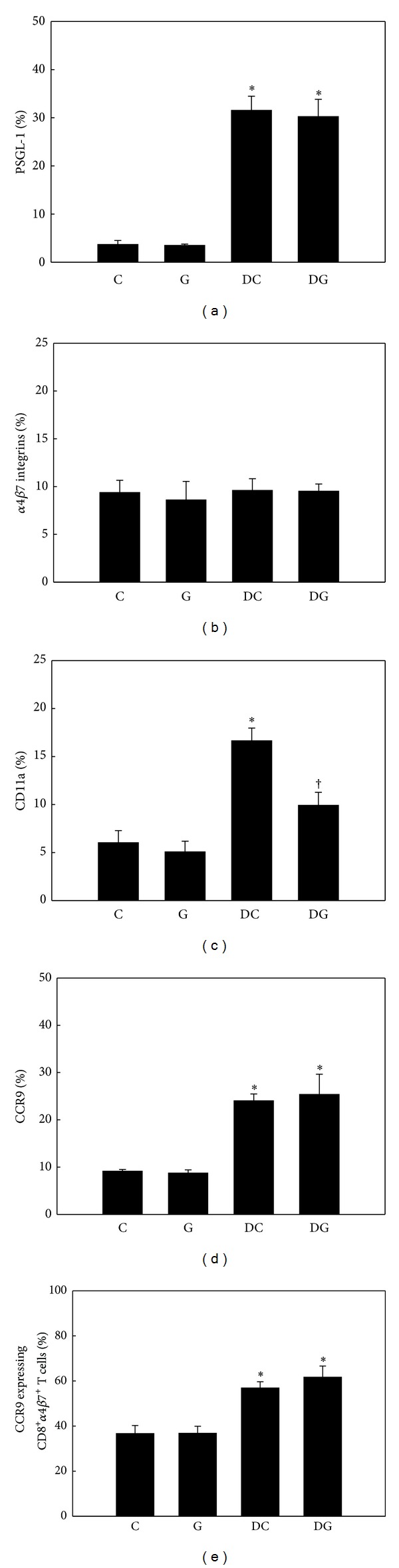
Percentage of adhesion molecule- and chemokine receptor-expressing cytotoxic T (Tc) cells in blood. CD8-positive blood lymphocytes were gated to analyze expressions of PSGL-1, *α*4*β*7 integrins, CD11a, and CCR9 by flow cytometry ((a)–(d)). (e) Expression of CCR9 by CD8 and *α*4*β*7 integrin double-positive blood lymphocytes. Values are shown as the mean ± SEM. *Significantly different from the C group (*P* < 0.05). ^†^Significantly different from the DC group (*P* < 0.05).

**Figure 7:**
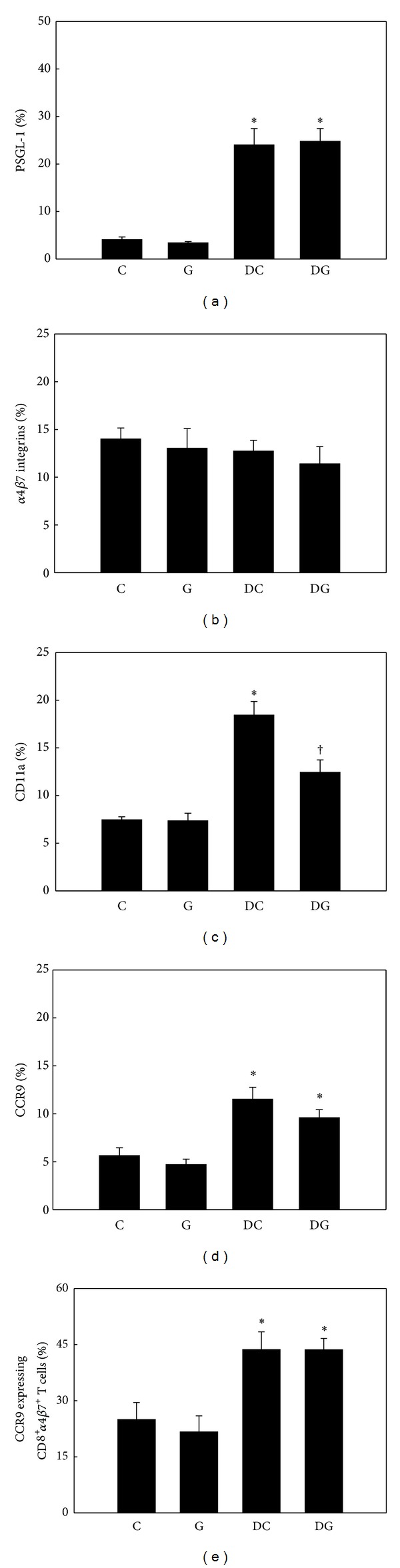
Percentage of adhesion molecule- and chemokine receptor-expressing cytotoxic T (Tc) cells in mesenteric lymph nodes (MLNs). CD8-positive MLN lymphocytes were gated to analyze expressions of PSGL-1, *α*4*β*7 integrins, CD11a, and CCR9 by flow cytometry ((a)–(d)). (e) Expression of CCR9 by CD8 and *α*4*β*7 integrin double-positive MLN lymphocytes. Values are shown as the mean ± SEM. *Significantly different from the C group (*P* < 0.05). ^†^Significantly different from the DC group (*P* < 0.05).

No differences in adhesion molecules and CCR9 expressed by blood and MLN Tc cells were observed between the C and G groups. The DSS colitis groups had higher percentages of PSGL-1-, CD11a-, and CCR9-expressing blood and MLN Tc cells, whereas expression levels of *α*4*β*7 integrins did not differ among the 4 groups. Compared to the DC group, the DG group had lower expression of CD11a by blood (Figures 6(a)–6(d)) and MLN Tc cells (Figures 7(a)–7(d)). There was no difference in expression levels of CCR9 on *α*4*β*7-positive Tc cells between the 2 DSS colitis groups (Figures 6(e) and 7(e)).

### 3.5. Gene Expression of Endothelial Adhesion Molecules in Colon Tissues

**Figure 8:**
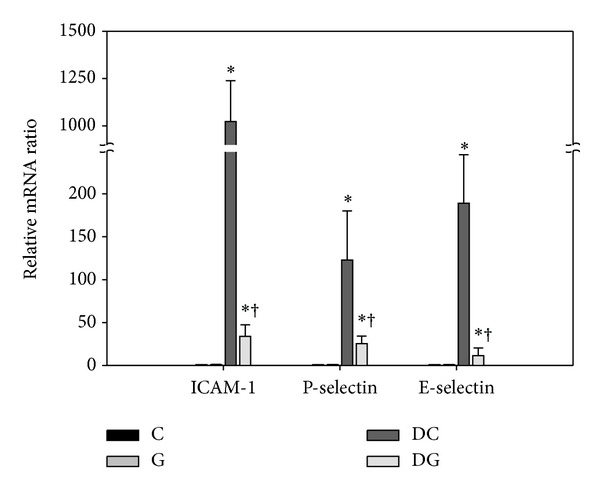
Gene expression of endothelial adhesion molecules in colon tissues. mRNA levels were analyzed by a real-time PCR. The C group was used as a calibrator, and the data were presented as the fold change in gene expression relative to the calibrator. Values are shown as the mean ± SEM. *Significantly different from the C group (*P* < 0.05). ^†^Significantly different from the DC group (*P* < 0.05).

There was no difference in mRNA levels of ICAM-1, P-selectin, and E-selectin between the C and G groups in colon tissues. DSS-induced colitis greatly upregulated the adhesion molecule genes expressed by activated endothelial cells. Compared to the DC group, the expression levels of ICAM-1, P-selectin, and E-selectin mRNA were suppressed in the DG group (Figure 8).

### 3.6. T Lymphocyte Subsets in the Colon Mucosa

**Figure 9:**
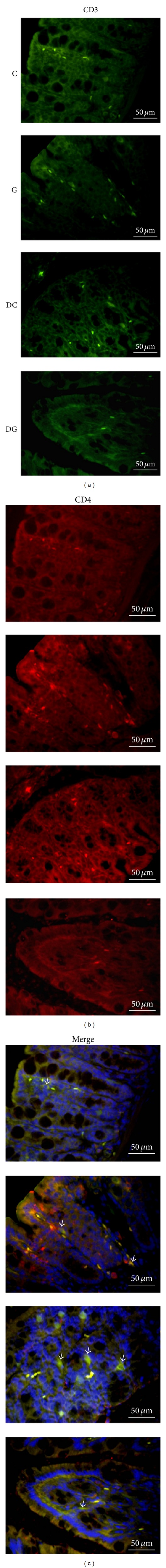
Representative examples of the immunofluorescence staining of T helper cells. In the left column, cell surface antigens were stained for CD3 (green, FITC), and staining of CD4 cells (red, rhodamine) is illustrated in the middle column. The nucleus is stained with DAPI (blue), and the last column presents a colocalized fluorescence image of CD3^+^CD4^+^ T cells.

**Figure 10:**
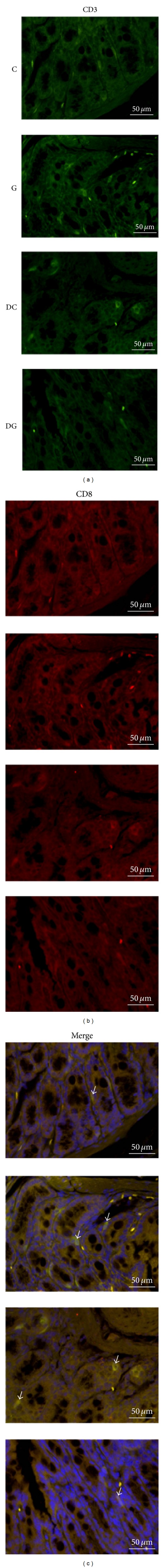
Representative immunofluorescence images of cytotoxic T cells. In the left column, cell surface antigens were stained for CD3 (green, FITC), and the staining of CD8 cells (red, rhodamine) is illustrated in the middle column. The nucleus is stained with DAPI (blue), and the last column presents a colocalized fluorescence image of CD3^+^CD8^+^ T cells.

CD3 is the cell surface marker of T cells. CD3 and CD4 double-positive cells are considered Th cells, whereas Tc cells coexpress CD3 and CD8. As shown in Figure 9, the immunoreactive intensity of Th cells was higher in the DC group than the DG group. However, intensities of Tc cell populations did not differ between the DC and DG groups (Figure 10).

## 4. Discussion

DSS is a heparin-like polysaccharide which results in acute chemical toxicity that disrupts the intestinal epithelial cell barrier [21]. DSS-induced colitis is characterized by extensive crypt and epithelial cell damage with ulceration, tissue edema, and infiltration of immune cells predominantly in the distal colon that mimics the histological features of UC [22]. Recent studies indicated that DSS-induced morphological and biochemical damage also extends to the small intestines [23]. Susceptibilities to DSS-induced colitis differ in various inbred mouse strains [24]. A single cycle of DSS exposure to the C57BL/6 strain was found to develop acute colitis which later proceeds to chronic inflammation, and T cell migration to the colon plays an important role in the progression to chronicity [25]. Regarding the high sensitivity to DSS, C57BL/6 mice were used in this study to analyze the consequences of DSS exposure on adhesion molecules and chemokine receptors involved in T cell trafficking to the intestines.

IBD is associated with a massive influx of immune cells into the gut. Previous studies indicated that increased local secretion of proinflammatory cytokines by the inflamed colon leads to upregulated expressions of vascular adhesion molecules, resulting in a sustained influx of inflammatory cells [26]. Although inflammation predominantly occurs in the GI tract, IBD patients are likely to develop extraintestinal manifestations [27] that may be attributed to aberrant activation and homing of T cells [28]. A recent study indicated that circulating CD4^+^ and CD8^+^ T cells are activated in both CD and UC patients, and these cells were correlated with leakage of microbial products from the impaired intestinal barrier [5]. Also, the severity of DSS-induced colitis was correlated with the immune response of MLNs [29]. A similar phenomenon was also observed in this study. Our results indicated that expressions of adhesion molecules and CCR9 on Th cells and Tc cells significantly increased in both blood and MLNs after DSS exposure. These findings suggest that both local and systemic T cells are activated. The role of Th cells in the pathogenesis of IBD has been widely studied. Dysregulation of Th cells can lead to immune cell infiltration into the intestinal mucosa and cause persistent inflammation [3, 6]. The pathogenic role of Tc cells in IBD has been less investigated. A previous study showed that antigen-specific Tc cells caused relapsing colitis in normal mice due to the cytolytic function against the intestinal epithelium [30]. Lee et al. [4] reported a gene expression profile of circulating CD8^+^ T cells that predicted a more aggressive disease course for IBD patients. However, the modulatory mechanism of Tc cells in IBD is still under investigation.

Naïve T cells constantly recirculate between the blood and secondary lymphoid organs. Once activated in secondary lymphoid organs, they become effector T cells that express adhesion molecules and chemokine receptors which control their extravasation into nonlymphoid tissue sites [31]. T cell trafficking to the gut and gut-associated lymphoid tissues (GALTs) requires *α*4*β*7 integrins. The ligand MAdCAM-1 is constitutively expressed by the mucosal endothelium in the small intestine and colon [32]. CCR9 is thought to participate in the specific localization of T cells to the small intestines because the sole ligand for CCR9, C-C chemokine ligand 25 (CCL25), is strongly expressed by the small intestinal epithelium [33]. However, Wurbel et al. [34] revealed that CCL25 expression increased during the recovery phase after acute DSS administration, suggesting a regulatory role of CCR9/CCL25 interactions during colonic inflammation. In this study, percentages of PSGL-1-, LFA-1-, and CCR9-expressing T cells were upregulated in acute DSS colitis, whereas expression levels of *α*4*β*7 integrins in colitic mice did not differ from those of normal mice. It is possible that T cell trafficking into the gut during acute DSS exposure is less dependent on *α*4*β*7 integrins. In support of our findings, Wang et al. [35] reported that localization of T cells to the intestines was relatively unaffected by *α*4*β*7 blockade during acute DSS-induced colitis.

Gln is a critical fuel source for enterocytes and immune cells. Gln supplementation attenuates gut injury by a complex mechanism, which involves protecting the epithelial barrier function, reducing oxidative stress, and modulating inflammatory responses [36]. The local and systemic immunomodulatory effects of Gln have been discussed in various experimental colitis models via different administration routes. Studies using rodents with trinitrobenzene sulfonic acid-induced colitis indicated that Gln given by the rectal route inhibits nuclear factor (NF)-*κ*B- and STAT-mediated inflammation in colon tissues [15] and further prevents colon fibrosis through downregulating gene pathways that contribute to the accumulation of matrix proteins [37]. We recently demonstrated that intraperitoneal pretreatment with alanyl-Gln, a Gln-containing dipeptide widely used in parenteral nutrition, suppresses cytokine expression in blood Th cells, reduces NF-*κ*B-mediated inflammatory responses in the colon, and upregulates expressions of genes which promote recovery of the colonic mucosa [17, 38].

Because most exogenous Gln is absorbed in the proximal small intestine, it might not reach the inflamed colon at a sufficient concentration to modulate inflammatory responses [36]. However, the enteral route of Gln administration still showed protective effects against DSS-induced damage. Oral Gln supplementation reduced the feces water content, enhanced expression of HSPs in the colonic mucosa, and ameliorated colon injury caused by DSS exposure [16, 39, 40]. A previous study indicated that oral Gln attenuated leukocyte adhesion and emigration in a rodent model of indomethacin-induced ileitis [41]. In this study, we demonstrated that oral Gln administration suppressed adhesion molecules and CCR9 expressed by T cells and downregulated the mRNA levels of adhesion molecules expressed by endothelium in colon tissues. The histological findings also support the results that Gln administration suppressed the infiltration of Th cells into the colon mucosa. Gln consumption is an important component of T cell activation [42, 43]. Different susceptibilities of Th and Tc cells to Gln supplementation may be explained by a higher cell population of Th cells, which competes as a Gln source with Tc cells. Further studies are needed to investigate the molecular mechanisms involved in gene expressions of adhesion molecules and CCR9 regulated by Gln.

In conclusion, this study showed for the first time that pretreatment with oral Gln reduced adhesion molecule- and CCR9-expressing T cells induced by DSS exposure. The inhibitory abilities against adhesion molecule and CCR9 expressions were more obvious in Th cells than Tc cells. Also, Gln supplementation reduced gene expressions of endothelial adhesion molecules in colons, prevented BW loss, and attenuated colon edema in colitic mice. Our results imply that dietary Gln prevented Th cell trafficking into colon tissues and provide a new mechanism of Gln supplementation that has beneficial effects on ameliorating the severity of acute DSS-induced colitis.

## Figures and Tables

**Table 1:** Composition of the semipurified diets.

Component	Control diet	Gln diet
	g/kg	
Soybean oil	100	100
Casein	200	150
Glutamine	0	41.7
Salt mixture^a^	35	35
Vitamin mixture^b^	10	10
Methyl cellulose	31	31
Choline bitartrate	2.5	2.5
Methionine	3	3
Corn starch	626.8	618.5

^a^The salt mixture contained the following (mg/g): calcium phosphate diabasic: 500; sodium chloride: 74; potassium sulfate: 52; potassium citrate monohydrate: 20; magnesium oxide: 24; manganese carbonate: 3.5; ferric citrate: 6; zinc carbonate: 1.6; curpric carbonate: 0.3; potassium iodate: 0.01; sodium selenite: 0.01; and chromium potassium sulfate: 0.55.

^b^The vitamin mixture contained the following (mg/g): thiamin hydrochloride: 0.6; riboflavin: 0.6; pyridoxine hydrochloride: 0.7; nicotinic acid: 3; calcium pantothenate: 1.6; D-biotin: 0.05; cyanocobalamin: 0.001; retinyl palmitate: 1.6; DL-*α*-tocopherol acetate: 20; cholecalciferol: 0.25; and menaquinone: 0.005.

**Table 2:** Leukocyte populations in blood and mesenteric lymph nodes (MLNs) (%).

	C	G	DC	DG
Blood				
Neutrophils	8.7 ± 0.5	8.0 ± 0.9	16.1 ± 2.7*	16.9 ± 2.1*
Monocytes	7.2 ± 0.9	6.7 ± 0.7	8.2 ± 1.3	7.7 ± 1.5
T cells	11.9 ± 0.4	11.7 ± 0.4	9.3 ± 1.2	9.3 ± 1.4
B cells	50.3 ± 0.9	53.4 ± 3.1	53.2 ± 2.7	49.0 ± 2.1
Th cells	6.7 ± 1.6	6.4 ± 0.4	7.3 ± 1.2	8.1 ± 0.8
Tc cells	3.6 ± 0.8	4.4 ± 0.3	4.5 ± 0.2	4.1 ± 0.3
MLNs				
T cells	45.2 ± 2.4	46.7 ± 2.2	32.1 ± 2.3*	34.9 ± 1.0*
B cells	30.3 ± 1.8	29.0 ± 3.3	31.3 ± 2.1	29.7 ± 1.4
Th cells	11.8 ± 0.3	12.3 ± 0.7	11.2 ± 0.9	12.1 ± 0.9
Tc cells	6.3 ± 0.4	6.7 ± 0.6	5.8 ± 0.3	6.5 ± 0.5

CD45-positive cells were considered to be leukocytes and gated to determine the population of leukocytes using a flow cytometer. Staining for Ly-6G, F4/80, CD3*ε*, and CD19 was used to respectively identify neutrophil, monocyte, T cell, and B cell populations. For the analysis of T cell subpopulations, lymphocytes were gated on the basis of their forward-and side-scatter profiles. Percentages of T helper (Th) and cytotoxic T (Tc) cells were respectively determined by CD4-and CD8-expressing cells in lymphocytes. Values are presented as the mean ± SEM. *Significantly differs from the C group (*P* < 0.05).
